# 4BR: Educational Training Programme for the Prevention of Sports Injuries in Young Athletes

**DOI:** 10.3390/ijerph18105487

**Published:** 2021-05-20

**Authors:** Joan Palmi, Nuria Alcubierre, Gonzalo Gil Moreno de Mora, Francesc Reig, Antoni Planas-Anzano

**Affiliations:** 1 National Institute of Physical Education of Catalonia, INEFC-University of Lleida, 25192 Lleida, Spain; ggilm@gencat.cat (G.G.M.d.M.); freig@gencat.cat (F.R.); aplanas@gencat.cat (A.P.-A.); 2 Avantmedic Center, 25008 Lleida, Spain; nurialcubierre@gmail.com

**Keywords:** recovery programme, sports injury, sports psychology, sports nutrition

## Abstract

This article provides a vision of the importance of the recovery process for the prevention of injuries in young athletes. From a sports psychology perspective, it presents a proposal for an optimisation programme to reduce the negative impact of exertion and subsequent risk of injury. The 4BR programme consists of three sub-programmes (technician advice, vulnerability detection, and the implementation of four recovery habit blocks). An interdisciplinary approach is taken to configure the four healthy blocks: nutrition–hydration, relaxation–rest, social life and personal moments. It demonstrates the importance of personalised adaptation to the sports context and moment in order to achieve maximum effectiveness of the proposed 4BR programme, which comprises workshops, exercises, evaluation systems and information feedback. The presented programme facilitates recovery, optimizes the return to training and reduces the risk of intrinsic injuries in young people. The conclusion drawn from the study is that there is a need to conduct further research to find empirical evidence of the positive effects of applying the 4BR programme to different sports.

## 1. Introduction

The aim of this study is to present a proposal for the prevention of training overloads by reducing the risk of intrinsic injuries in young athletes through the 4BR programme [1] (4BR; according to original acronyms in Spanish [1]). The prevention of sports injuries should be a major object of research, because it is evident that the situation regarding injuries in young athletes is not improving, despite the considerable work being done. There is a need not only to explore issues beyond the risk factors and generic injury mechanisms, but also to expand our understanding of the complex interactions of the contextual factors influencing injuries. We highlight the importance of the psycho-educational process in recovering from effort made, knowing that athletic performance requires a variety of explosive and demanding motor actions in order to achieve optimal performance, which begins in young people and reaches its peak in adulthood. This requirement implies high resistance and, predominantly, anaerobic power, which often lead to injuries that, when occurring in sport, are called sports injuries. These are usually related to the fatigue perceived by the athlete [2,3], which manifests itself as a loss of vigour, a feeling of weakness, and greater vulnerability to injury.

It is known that exercise in adolescence and youth, which includes Physical Education (PE) in the school environment as well as recreational and competitive sports, is very important to developing habits that not only affect the health of those participating in it, but also have vital significance in adulthood. Youth is the stage of the life cycle in which the maximum rate of growth in stature is reached. This process of the musculoskeletal system’s physical transformation directly affects a gradual process of physical, cognitive, emotional and social changes [4].

According to the results of a study conducted by our group [5], motivation is a key component of doing physical exercise. Fun and well-being help individuals stick to regular and continued exercise. For this reason, adapting the role of PE and training to the interests of young athletes/non-athletes will help them to complete adequate physical/sports activities almost daily, which prevents the observed deterioration in physical condition (flexibility, cardiovascular resistance and strength) in young people [6]. In the specific process of applying repeated training loads, or when competitions come one after the other, recovery time and quality may not be respected (insufficient rest) and a decrease in performance may be observed (negative overcompensation), thus developing into overtraining syndrome [7] and subsequent injuries. The individual consequences that result from these overuse injuries in the short, medium or long term may not only lead to a loss of training or competitions, but at these ages it can also have a severe impact on daily personal activities (family, friends, studies, etc.), thus creating a significant state of personal anguish and anxiety that may even become a potential cause of giving up sport entirely.

This issue of sports injury is addressed from a psychological perspective, thus falling within a framework of sports psychology with a preventative outlook and pain-suffering reduction [8]. The specialisation of each sport, and age, gender, performance level and organisational culture [9] are taken into account, and a multidisciplinary perspective is adopted, with the conviction that the psychological components should be known, evaluated and incorporated into every injury process, both in prevention [10,11,12] and post-injury rehabilitation [13,14,15,16,17,18,19].

This article presents an intervention proposal for sports injury prevention through an integrated educational training programme for the post-effort recovery period.

## 2. Prevention Proposal: The 4BR Programme

From sports injury psychology perspective, prevention work has several objectives: technician advice/awareness, assessment of risk of injury due to psychosocial factors (vulnerability), and preventative/rehabilitative intervention in injuries. Our group has been working for two years on a prevention model that attaches great importance to the training of athletes in improving habits in the recovery (4BR programme [1]). The proposal is based on the work of Kenttä and Hassmén [20] for the prevention of overload and overtraining syndrome [7], reducing the risk of injury due to internal causes.

The 4BR programme contains three sub-programmes; technician advice, athlete vulnerability detection, and the implementation of recovery habits (Table 1). Each sub-programme is carried out in several workshop sessions, with the latter of the three specifically focusing on content development for improving recovery habits (periods of non-physical work). The three aforementioned sub-programmes are developed continuously.

Sub-programme 1: Technician advice. This section aims to raise awareness and involve the coaching staff in the need to know the risk factors of both internal (personality, anxiety, daily stressors, injury history) and external factors that predispose to injury. In this first multi-professional meeting, it is necessary to nominate a named person to take responsibility for the detection and control of the external factors (to check equipment, facilities, etc.). The importance of habits in the recovery process of young athletes will also be raised. The four recovery blocks provided for in the 4BR programme are presented. It also sets out the commitment to provide feedback on the information obtained from the evaluation systems to both the athletes and the coach.

Sub-programme 2: Athlete vulnerability detection. This includes the assessment systems that are applied to athletes to assess their risk of injuries due to internal predisposition (neuroticism, impulsivity, coping, competitiveness, motivation oriented to success, distress tendency, history of stressors, injury history, burnout and other states). The factors are evaluated in accordance with the requirements of validity and reliability, with the ethical commitment to provide the athletes with feedback on the results so that they are aware of their vulnerability to injury.

**Figure 1:**
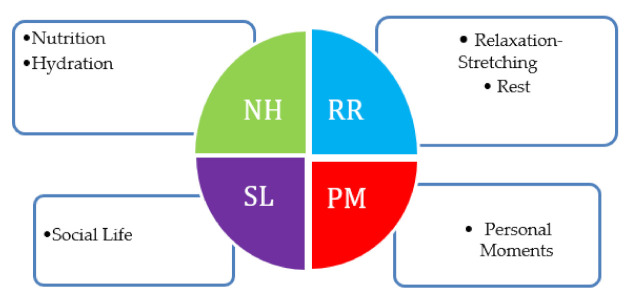
Representation of the four recovery habit blocks.

Sub-programme 3: Implementation of recovery habits. The intervention programme content includes the improvement of sports recovery behaviours and the adjustment of habits through psycho-educational work to achieve maximum athlete self-regulation [21]. Shown below are the four blocks relating to the implementation of the recovery habits sub-programme. (see Figure 1):

### 2.1. Nutrition and Hydration Block (NH)

Given the characteristic factors of young amateur athletes, the energy and water requirements must be analysed. The correct assessment is of vital significance in their maturational stage [22,23,24]. The establishment of optimal qualitative and quantitative nutritional guidelines, as well as proper periodisation, allows important objectives to be achieved, such as growth curve optimisation, performance, recovery, health, body composition, training adaptation, and risk-of-injury minimisation [25]. Current knowledge in the area of recovery emphasises the importance of nutritional education in adolescence as a risk or protection factor against certain objectively evaluable results, such as performance optimisation, intra- and inter-session post-effort recovery, musculoskeletal pathology or sports injury prevention, and intake adequacy [26].

**Figure 2:**
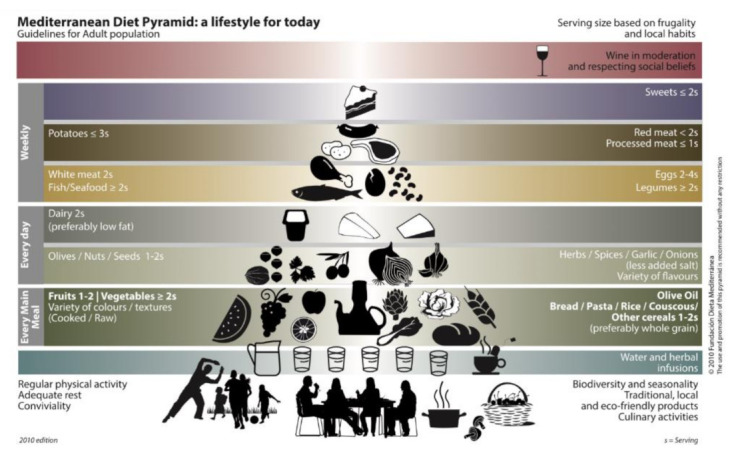
Mediterranean Diet Pyramida.

The traditional Mediterranean diet (MedDiet) [27,28], is one of the best food options during adolescence, and at any stage of life [28,29]. The MedDiet constitutes an ideal eating pattern for the preservation of state of health, quality of life and ideal weight (Figure 2). It involves a considerable consumption of vegetables, fruits, whole grains, legumes, bread and olive oil, and a moderate consumption of meats and fermented lactic products, both within a strongly socialising context, combined with regular physical activity [29] and good hydration. Recent research shows that, during adolescence, there is less adherence to an adequate eating pattern, while risk behaviours, the need for socialisation and the tendency towards fast-food consumption are greater [30,31]. Therefore, we consider that the traditional MedDiet, as an inherent part of the 4BR model, has a positive influence on the important construct of health-related quality of life (HRQoL) during this stage, thus highlighting its multidisciplinary nature and emphasising the safeguarding spirit of a healthier eating pattern.

### 2.2. Relaxation-Stretching and Rest Block (RR)

Reducing pain caused by exertion and improving the perception of well-being lead to a decrease in specific risk of injury [32,33]. Thus, highlighted in this block is the need to: (1) stretch the different muscle groups for 10–15 min immediately after effort, (2) reduce body temperature by taking a good shower, and (3) perform conscious breathing 1.5–2 h after finishing training. During the implementation process, specific relaxation exercises are carried out (coordinated with the physical trainer). In this section, work is also carried out on the importance of night-time and after-lunch rest. The aspects considered are the right amount of restorative sleep for this age group (around 8 h), having pre-sleep habits (having dinner an hour and a half before going to bed, restricting the use of technological devices, such as smartphones, tablets, etc., for 30 min before going to sleep, as well as remembering to consciously carry out hygienic habits). Resting for 20 min after every meal to help with digestion and well-being is also considered. We emphasise aspects of the compensatory recovery period from the physical work carried out, especially in young people experiencing the time pressures that a dual study and sport career puts on them [34].

### 2.3. Social Life Block (SL)

This block refers to the importance of taking care of the psychosocial component of a young athlete’s environment. The importance and self-analysis of the personal (family), educational (studies), sports and socio-cultural environments should be included. The aim is to ensure that the athlete is aware not only of the importance of his or her position in relation to the environment, but also of interpersonal relationships for well-balanced development, and of enhancing daily/weekly contacts within his or her environment, thereby improving the perception of social support that facilitates personal well-being [34]. The athlete is advised to analyse the time he or she spends with his or her non-athlete friends or fellow athletes; to make a list of his or her best friends and assess the time he or she spends with them in a week; and to assess the family relationship and the time spent on it, and how it relates to his or her personal context. The importance of both face-to-face and virtual contacts through phone and social networks is reaffirmed. Finally, socio-cultural activities and the time spent on them in a month are analysed (cinema, theatre, concerts, youth clubs, helping the community, etc.).

### 2.4. Personal Moments Block (PM)

The importance of daily moments when a young athlete relates to his or her body is clarified, along with his or her most personal needs that help him or her regain calm after the effort and daily stressors. The athlete is advised to write and comment on moments when he or she consciously feels at ease, and to notice the recharge process that compensates for the effort made (e.g., “It’s my turn now”; “This is my time!”). The importance of time management throughout the day is analysed, since one of the most stressful factors for a young athlete is a dual study and sport career [35,36], which may lead him or her to give up sport as a result of burnout, an issue about which awareness must be raised [37]. He or she is made aware of this dual exigency, and of the need to have a planned schedule to balance the times spent on each aspect, and ideas are suggested, such as keeping a weekly diary/calendar, and also daily compensatory aspects such as taking care of personal image (time to buy clothes), the importance of smells in their immediate environment, personal hygiene, self-massage in the areas most affected by doing sport, use of protective-moisturising creams, listening to music, controlled time spent on hobbies, etc.

These habits must be implemented and evaluated, as indicated in the next section.

## 3. Plan Proposal

A valid generic plan for all young athletes is extremely difficult to achieve due to the great diversity of personal situations and commitments. These contexts are conditioned by factors such as the number of competitions and training sessions, sports season, educational activities (lessons, exam periods, etc.), and personal commitments (activities with family, friends, etc.). This affects the situation of a young athlete, who generally has little time available and limited personal predisposition. However, to develop the 4BR programme, an individualised plan will be necessary according to individual sports group-level, specialist psychologist role and sport characteristics [38,39]. Described below are the different tools for use when developing a plan based around workshops, exercises, evaluation systems and information feedback.

Technician advice sub-programme: Workshops for the technical staff. Two to three training sessions, lasting approximately 20 min each, to raise awareness of an athlete’s own risks (personality, daily stressors, experience) and external risks (demand, overloads, inappropriate equipment). The 4BR project is also presented, showing not only the importance of habits for a good recovery, but also the plan proposal, requesting the collaboration and involvement of the coaching staff. Finally, a feedback session is added, for the evaluation of athletes and of the project process.

Athlete vulnerability detection sub-programme: Evaluation of athletes. Data collection within a framework of not burdening athletes with paper and pencil tests, and of trying to present a certain variety that may be more attractive. We propose mood, anxiety, recovery assessment, perception of effort, perception of compliance with the four recovery habit blocks, and a final evaluation of the 4BR programme.

Implementation of the recovery habits sub-programme: Workshops for athletes. Two to three training sessions, lasting approximately 20 min each, on the importance of well-being and recovery periods, and presentation of the 4BR programme; and four to five sessions of exercises for athletes, lasting 20 min each, where exercises are presented and training given to improve activation (abdominal breathing rhythm, progressive muscle relaxation, stretching, respiratory and body awareness) and group cooperation–bonding (team-building exercises).

Information feedback: One to two individual sessions in which athletes are informed of the evaluation results and of their progression on the 4BR programme, and individualised coping strategies are suggested.

## 4. Conclusions

Reducing the risk of injury in young athletes is of crucial importance to sports organisations and professionals. However, there are few psychological interventions for sports injury prevention [38,39,40]. We know that injuries reduce the athletes’ well-being and development, and lead to a significant loss of sports potential. Further research is therefore required to validate empirical evidence of the 4BR programme’s application to different sports. Thus far, the programme has been applied to soccer, roller hockey and basketball, with positive results for both athletes and coaches, but its quantitative evaluation has been hindered by the impact of the COVID-19 pandemic (currently in the process of conducting the empirical study for the 2020–21 season).

To ensure the programme is adequately implemented, we would underscore the need to properly define the objectives and the interventions so that they can be applied correctly to the sports context. This will allow them to be easily adapted, validated and replicated by different professionals with an interdisciplinary vision. By achieving a reduction in post-sport effort fatigue, a good perception of well-being, a reduction in overtraining syndrome and a reduction in injuries due to intrinsic causes, young athletes will be the ultimate winners [41,42,43].

## Figures and Tables

**Table 1:** Sub-programmes of the 4BR proposal (four recovery blocks) for the prevention of sports injury.

1. Technician advice sub-programme
Sensitivity to risk factors (internal and external).
b. Importance of habits in the post-effort recovery process (presentation of the four recovery habits’ content).
c. 4BR evaluation feedback.
2. Athlete vulnerability detection sub-programme
Personal predisposing factors (personality, anxiety, daily stressors).
b. History of injuries.
3. Implementation of recovery habits sub-programme
Presentation of the four habit blocks.
b. Implementation and evaluation of the four habit blocks.
